# Mapping the immune microenvironment of *KIT*- and *PDGFRA*-mutant GISTs reveals genotype-specific immune infiltration and TKI-induced remodeling

**DOI:** 10.3389/fimmu.2026.1961524

**Published:** 2026-10-15

**Authors:** Alice Costa, Maria Concetta Nigro, Marco Grillini, Livia Gozzellino, Margherita Nannini, Ilenia Motta, Antonio De Leo, Maria Giulia Pirini, Massimo Del Gaudio, Matteo Ravaioli, Manuela Ianni, Gianandrea Pasquinelli, Annalisa Astolfi, Maria Abbondanza Pantaleo

**Affiliations:** 1 IRCCS Azienda Ospedaliero-Universitaria di Bologna, Bologna, Italy; 2 Medical Oncology, IRCCS Azienda Ospedaliero-Universitaria di Bologna, Bologna, Italy; 3 Pathology Unit, IRCCS Azienda Ospedaliero-Universitaria di Bologna, Bologna, Italy; 4 Department of Medical and Surgical Sciences (DIMEC), University of Bologna, Bologna, Italy; 5 General Surgery and Transplant Unit, IRCCS, Azienda Ospedaliero-Universitaria di Bologna, Bologna, Italy

**Keywords:** digital pathology, gastrointestinal stromal tumors, imatinib, immunohistochemistry, immunomicroenvironment, KIT, PDGFRA, RNA sequencing

## Abstract

**Background:**

GIST tumor microenvironment is characterized by an abundant immune infiltrate. Imatinib exerts immunomodulatory effects that may contribute to a more immunosuppressive microenvironment. Nonetheless, checkpoint inhibitors have demonstrated limited efficacy to date supporting the rationale for combination strategies integrating immunotherapy and TKIs to enhance anti-tumor immune response. This study aims to identify immunological predictive biomarkers and to establish a rational framework for a personalized immunotherapeutic strategy capable of synergizing with TKIs in advanced GIST.

**Materials and methods:**

We performed an integrated immune profiling of 49 *KIT-PDGFRA*-mutant GIST samples. Multiplexed immunohistochemistry was used to quantify innate and adaptive immune populations, regulatory subsets, immune checkpoint expression, HLA class I/II status. Immune features were assessed by digital pathology and analyzed according to genotype, site, primary versus metastatic disease, TKI exposure. In a subset of tumors, bulk RNA sequencing and CIBERSORTx deconvolution were used to complement tissue-based immune characterization.

**Results:**

GISTs displayed a heterogeneous but consistently immune-infiltrated microenvironment enriched in CD163-positive M2-like tumor-associated macrophages. T cells represented the second largest immune population and included both CD8+ and CD4+ T cells. NK cells were also detectable, whereas B cells and regulatory T cells were generally less abundant. CTLA-4 expression was widespread but low, whereas tumor-cell PD-L1 expression was highly variable. Notably, complete or subclonal HLA class I loss was identified in 20% of tumors. *PDGFRA*-mutant GISTs showed a significantly inflamed profile, with increased B and T lymphocytes and NK cells. Intestinal GISTs exhibited higher infiltration by T-cell, NK and macrophage populations than gastric tumors. In *KIT*-mutant primary tumors, imatinib exposure markedly reshaped the immune microenvironment, reducing regulatory T cells, NK cells and CD68/CD163-positive macrophages while relatively preserving CD8-positive T cells.

**Conclusions:**

GISTs harbor a rich and heterogeneous immune microenvironment shaped by genotype, site, TKI exposure, antigen-presentation status. The complete or subclonal HLA-I loss as a potential immune escape mechanism, a more lymphoid-, B-cell-, and NK-enriched immune profile in *PDGFRA*-mutant tumors, the site-specific immune enrichment of intestinal GISTs, and the selective remodeling of regulatory and myeloid immune compartments after imatinib refine the current understanding of GIST immunobiology and support the development of rational, biomarker-driven immunotherapeutic strategies.

## Introduction

Gastrointestinal stromal tumors (GISTs) are mesenchymal malignancies of the digestive tract characterized by mutually exclusive activating mutations in genes encoding receptors of tyrosine kinases, most commonly *KIT* (~70-80%) or *PDGFRA* (5-15%), leading to constitutive activation of downstream proliferative signaling pathways (1). Approximately 10-15% of GISTs lack canonical *KIT* or *PDGFRA* alterations and harbor different oncogenic drivers, including *NF1* or *BRAF* mutations (~10%), succinate dehydrogenase (SDH) complex deficiency (<5%), and rare gene fusions (<1%) (2).

The introduction of tyrosine kinase inhibitors (TKIs), such as imatinib, has revolutionized the therapeutic landscape of GISTs (3). However, the long-term efficacy of TKIs is limited by the development of acquired resistance, primarily driven by secondary kinase mutations, as well as by intratumoral polyclonal heterogeneity evaluated also in ctDNA studies (4–6). Current clinical management is therefore based on a sequential therapeutic strategy involving subsequent lines of therapy, including sunitinib, regorafenib and ripretinib, while avapritinib has displayed remarkable efficacy in *PDGFRA* D842V-mutant cases (7).

A central aspect of overcoming TKI resistance is the comprehensive characterization of the tumor microenvironment (TME), which plays a critical role in disease progression, immune evasion, and therapeutic resistance (8). Although GISTs have long been considered neoplasms with a low tumor mutational burden (TMB) and therefore regarded as poorly immunogenic, recent evidence based on whole-exome sequencing has revealed a significant genetic heterogeneity, capable of generating somatic neoepitopes (9–11). The prediction and functional validation of such neoantigens are of particular relevance, as even single oncogenic mutations have been shown to generate neoepitopes with high affinity for HLA molecules, potentially rendering tumor cells recognizable by the immune system (9).

The GIST TME exhibits distinctive immunological features compared with those of epithelial malignancies. Analyses based on immunohistochemistry (IHC), flow cytometry, and more recently single-cell transcriptomics have shown that GISTs are immunologically rich tumors, characterized by an abundant immune infiltrate, with tumor-associated macrophages (TAMs) as the predominant immune population, followed by T lymphocytes (CD4+, CD8+) and natural killer (NK) cells (12–14). Nevertheless, the TME remains largely immunosuppressive. TAMs frequently display a hybrid polarization state, co-expressing both pro-inflammatory (M1) and immunosuppressive (M2) signatures, a phenotype that has been associated with tumor progression and impaired antigen presentation (14). Furthermore, tumor-infiltrating CD8+ T cells often exhibit features of functional exhaustion, driven predominantly by LAG-3 expression (8, 14). Moreover, immunological features appear to vary according to the mutational genotype, suggesting that distinct oncogenic drivers (e.g., *PDGFRA* vs *KIT*) may shape divergent tumor-immune profiles (9, 15).

TKI therapy further complicates the immunological landscape. Imatinib not only inhibits oncogenic signaling but also exerts relevant immunomodulatory effects, including modulation of NK cell activity, downregulation of immunosuppressive enzymes such as indoleamine 2,3-dioxygenase (IDO) and polarization of tumor-associated macrophages (TAMs) toward M2-like states, with a concomitant reduction in intratumoral inflammatory cytokines (8, 14, 16). Collectively, these effects may contribute to a more immunosuppressive TME.

Despite these observations, immune checkpoint inhibitors (such as anti-PD-1 or anti-CTLA-4 antibodies) have demonstrated limited clinical efficacy to date, supporting the rationale for combination strategies integrating immunotherapy and TKIs to enhance anti-tumor immune response and overcome resistance (17, 18). Further investigation into the integration of TKI inhibition and immunotherapy, based on a comprehensive characterization of the mutational landscape and the tumor immune microenvironment, is needed. By analyzing the tumor mutational status and the cellular composition of the TME, this study aims to identify immunological predictive biomarkers and to establish a rational framework for the development of personalized immunotherapeutic strategies capable of synergizing with TKIs in the treatment of advanced GISTs.

## Materials and methods

### Patients and tumor samples

A total of 49 GIST tumor samples retrospectively collected were analyzed. The aggregate tumor and clinical features of the cohort are reported in **Table 1**. The tumor and clinical characteristics of all patients are reported in **Supplementary Table 1** (19). All samples were characterized by *KIT* or *PDGFRA* mutations, and *KIT*/*PDGFRA* WT GIST cases were excluded. 42 samples were from primary tumors and 7 from metastases. 18 patients received therapy with TKIs, 15 of them received imatinib and 3 imatinib and sunitinib. This study was performed in agreement with the Declaration of Helsinki and approved by the local institutional ethics committee of IRCCS Azienda Ospedaliero-Universitaria Policlinico S.Orsola-Malpighi (approval number 113/2008/U/Tess). All patients provided written informed consent.

**Table 1:** GIST tumor and clinical characteristics.

N tot 49 pt	Median (range) vv
**Age**	58 (34–85)
**Gender**	**N (%)**
Female	26 (53.1)
Male	23 (46.9)
Primary tumor site	
Stomach	33 (67.3)
Duodenum	2 (4.1)
Jejunum	4 (8.2)
Ileum	9 (18.4)
Colon	1 (2.0)
Disease status diagnosis	
Localized	43 (87.8)
Advanced	6 (12.2)
Risk Stratification	
Null	3 (6.1)
Very low	6 (12.2)
Low	7 (14.3)
Intermediate	7 (14.3)
High	16 (32.6)
Not evaluable	4 (8.2)
Not available	6 (12.2)
Mutation	
KIT	40 (81.6)
PDGFRA	9 (18.4)
Tissue sample	
Primary	42 (85.7)
Metastases	7 (14.3)
Pre-treated	
Yes	18 (36.7)
No	31 (63.3)

### Immunohistochemistry

Consecutive 3-μm-thick formalin-fixed paraffin-embedded (FFPE) tumor sections were used for immunohistochemical analysis. Single and dual immunohistochemical staining procedures were performed on a BenchMark ULTRA platform, while triple multiplex staining was performed on a DISCOVERY ULTRA platform (Ventana Medical Systems/Roche Diagnostics, Tucson, AZ, USA), according to optimized laboratory protocols.

The immune panel included single immunohistochemical stainings for Arginase-1, HLA class I (HLA-I) and HLA class II (HLA-II); dual chromogenic stainings for CTLA-4/NCR1 and PD-L1/CD68; and triple stainings for CD3/CD20/CD163 and FOXP3/CD8/CD4. In the dual stainings, CTLA-4 and PD-L1 were visualized using DAB, while NCR1 and CD68 were visualized using a red chromogen. In the CD3/CD20/CD163 multiplex panel, CD3 was visualized in purple, CD20 in yellow, and CD163 in teal. In the FOXP3/CD8/CD4 multiplex panel, FOXP3 was visualized using DAB, CD8 in purple, and CD4 in yellow. FOXP3+/CD4+ cells were interpreted as cells with a regulatory T-cell (Treg) phenotype. In our cohort, the staining patterns were generally well defined, with no cases showing expression levels close to the thresholds used for categorical classification. Nevertheless, all cases were independently reassessed by a second pathologist, blinded to the original evaluation.

Details on antibody clones, antigen retrieval conditions, incubation times, detection systems, and chromogenic substrates are provided in **Supplementary Tables 2A, B**.

### Slide scanning and digital pathology workflow

All stained slides were digitized at 20× magnification using a Ventana DP600 slide scanner (Ventana Medical Systems/Roche Diagnostics). Whole-slide images (WSIs) were imported into QuPath version 0.5.1 for digital image analysis (20).

For each case and staining, the tumor area was identified using a supervised pixel classifier trained to distinguish tumor tissue from non-tumor tissue, including normal tissue, background, necrosis, artifacts, and other non-informative regions. The resulting tumor annotations were reviewed and, when necessary, manually refined to ensure accurate exclusion of non-representative areas before downstream cell-based analysis.

Cell detection was performed within the tumor annotations using a dedicated StarDist-based workflow implemented in QuPath through customized Groovy scripts (21). Detection parameters were optimized for chromogenic multiplex brightfield images and kept consistent within each staining panel. Following nuclear/cellular segmentation, marker-specific measurements were extracted for each detected cell.

Object classifiers were then trained in QuPath to assign each cell to the corresponding immune or tumor cell category based on chromogen-specific signal. For the CD3/CD20/CD163 panel, cells were classified as CD3-positive T cells, CD20-positive B cells, CD163-positive macrophages, or marker-negative tumor cells. For the FOXP3/CD8/CD4 panel, cells were classified as CD8-positive T cells, CD4-positive T cells, CD4+/FOXP3+ cells with a regulatory T-cell phenotype, or marker-negative tumor cells. For the CTLA-4/NCR1 and PD-L1/CD68 dual stainings, object classifiers were trained to recognize CTLA-4-positive cells, NCR1-positive cells, CD68-positive macrophages, and PD-L1-positive tumor cells, as appropriate for each panel.

The final classifications were visually reviewed on representative regions to confirm adequate correspondence between chromogenic signal and object class assignment.

### Marker assessment and quantification

Arginase-1, HLA-I, and HLA-II expression were assessed by two expert pathologists within the annotated tumor area (A.D.L. and M.G.P.) on WSIs. For HLA-I, tumor-cell expression was categorized as retained, subclonal loss or loss using previously described criteria (22), while for HLA-II it was classified as absent or subclonal. Retained expression was defined as staining in more than 90% of tumor cells, subclonal expression/subclonal loss as staining in 10–90% of tumor cells, and loss/absent as staining in less than 10% of tumor cells. Arginase-1 expression was recorded as present or absent, with absence defined as no detectable specific signal. Appropriate internal positive controls were considered. Complete inter-observer agreement was observed for HLA-I and HLA-II classification (Cohen’s κ = 1.00). Complete agreement was also observed for Arginase-1 assessment, with both observers confirming the absence of positive staining in all cases.

All remaining immune markers were quantified digitally using QuPath (**Figure 1**). For each marker, immune cell infiltration was expressed as both cell density (positive cells/mm²) and percentage of positive cells among all detected cells within the tumor annotation. PD-L1 expression was quantified as the percentage of PD-L1-positive tumor cells among all detected tumor cells, corresponding to the tumor proportion score (TPS). Cases with a TPS ≥1% were considered PD-L1 positive, according to the threshold previously reported by Sun et al. (14). In addition, the combined positive score (CPS) was calculated as the number of PD-L1-positive tumor cells, lymphocytes, and macrophages divided by the total number of viable tumor cells and multiplied by 100.

**Figure 1:**
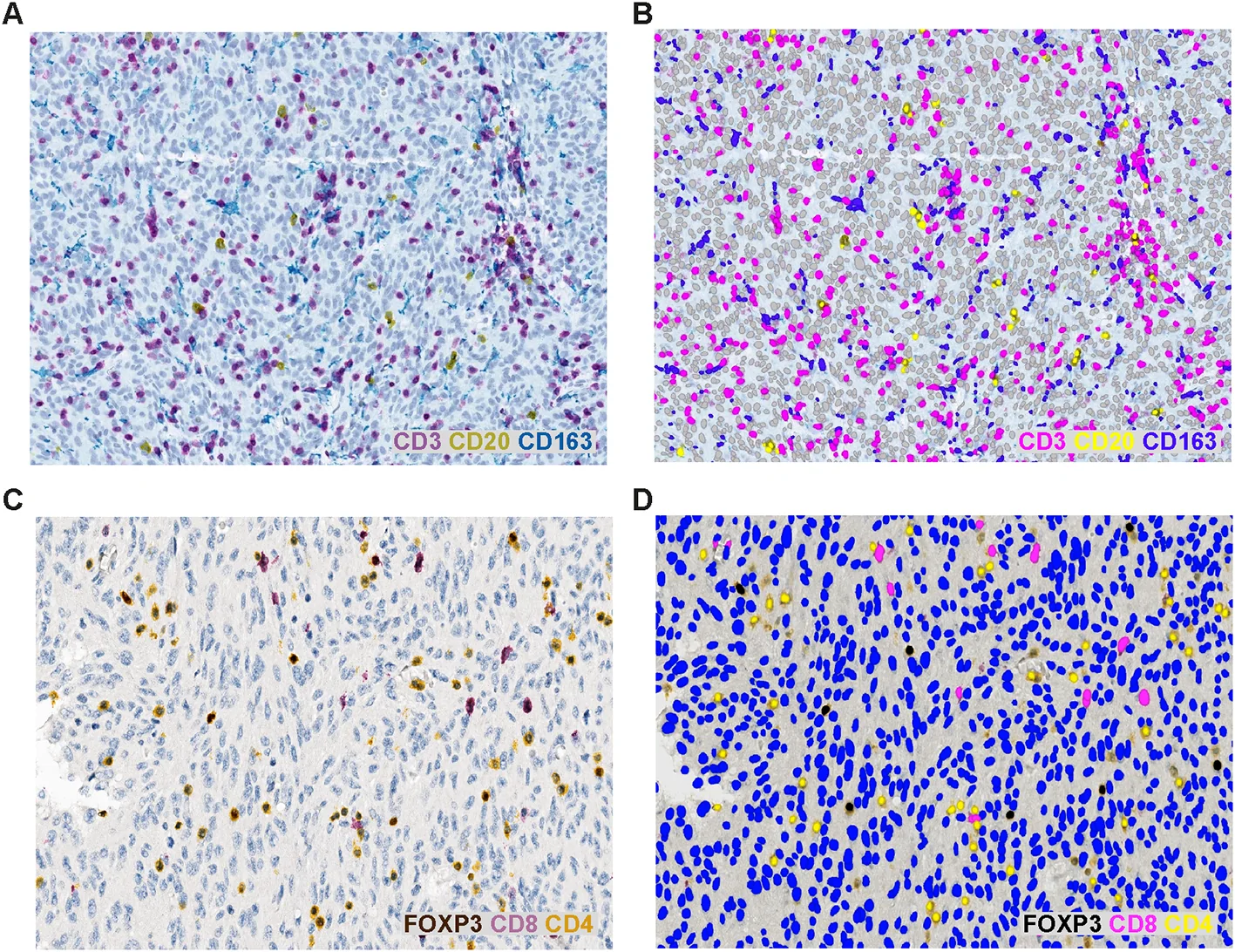
Triple multiplex IHC.Original image and corresponding QuPath cell detection for CD3/CD20/CD163 **(A, B)** and for FOXP3/CD8/CD4 **(C, D)**. In **(B)**, detected cells are color-coded as follows: CD3, purple; CD20, yellow; CD163, teal; tumor cells, gray. In **(D)**, detected cells are color-coded as follows: FOXP3, black; CD8, purple; CD4, yellow; tumor cells, blue. Magnification: 20× **(A, B)** and 40× (**C, D**).

### RNA-sequencing and bioinformatic analysis

RNA-sequencing was performed on a subset of samples selected based on tissue availability and RNA quality. Firstly, RNA was extracted from FFPE slides using the RecoverAll Total Nucleic Acid Isolation Kit (Invitrogen, Thermo Fisher Scientific, Waltham, MA, USA). Libraries were obtained from 10 ng of total RNA using the SMART-Seq Stranded Kit (Takara Bio USA Inc., San Jose, CA, USA), following the manufacturer’s protocol. Sequencing was performed on Illumina NextSeq 500 and 2000 (Illumina, San Diego, CA, USA).

A primary quality control of all the FASTQ files was performed by FastQC and MultiQC, followed by the removal of low-quality regions by fastp (23, 24). Subsequently, reads were mapped on the reference human genome hg38 adopting STAR, while BAM files were sorted and indexed by SAMtools (25–27). FeatureCounts was applied to obtain raw gene counts, which were then normalized as transcripts per million (TPM) (28). TPM were used to estimate the absolute and relative abundance of immune populations in each sample via CIBERSORTx, using the LM22 signature matrix (29).

### Statistical analysis

Statistical analysis were performed using GraphPad Prism version 8 (GraphPad Software, San Diego, CA, USA). The distribution of continuous variables was assessed using the Shapiro-Wilk normality test, and the corresponding *p*-values are reported in **Supplementary Table 3**. Data are presented as means with standard deviations (SDs) for normally distributed variables and as median values with interquartile ranges (IQRs) for non-normally distributed variables. Comparisons between two independent groups were performed using Welch’s unpaired *t-*test for normally distributed variables and the Mann–Whitney U test for non-normally distributed variables. For Mann-Whitney U comparisons, the magnitude of the between-group difference was estimated using the Hodges-Lehmann estimator, whereas for Welch’s *t-*test comparisons it was expressed as the difference between means. The corresponding 95% confidence intervals are reported in **Supplementary Table 3**. To account for the assessment for multiple immunological markers, raw two-sides *p*-values were adjusted using the Benjamini-Hochberg procedure within each family of comparisons to control the false discovery rate (FDR) at 5%. Raw *p*-values and the corresponding FDR-adjusted *q*-values are reported in **Supplementary Table 3**. A two-sided p-value <0.05 was considered statistically significant (significance is indicated as *p < 0.05, **p < 0.01, and ***p < 0.001).

## Results

A comprehensive characterization of the mutational status and the tumor immune microenvironment was performed in a series of 49 *KIT-* or *PDGFRA*-mutant GIST tumor samples, both overall and according to pathological and clinical features.

### Composition of the immune infiltrate across the GIST cohort

Characterization of the GIST TME (**Figure 2A**) revealed a marked enrichment of macrophages, with CD68-positive (CD68+) cells representing the predominant immune population. Among these, the M2-like macrophages (CD163+) were the main component of the tumor-associated immune infiltrate. CD3+ T lymphocytes constituted the second major immune population, with a relative enrichment of CD8+ cytotoxic T lymphocytes compared with CD4+. The positivity of NCR1 supported the presence of NK cells within the TME. In contrast, B cells were consistently detected at lower levels across the cohort, based on the positivity of CD20. Indeed, Tregs (CD4+/FOXP3+) represented a significant proportion of CD4+ T cells, accounting for an average of 25% of the T-helper population (range: 3.5-100%). Immune checkpoint expression was scattered across the series (**Figure 2B**), with CTLA-4 being positive in all samples with a low median density (0.68; range: 0.04-8.97). Tumor-cell PD-L1 expression, assessed by TPS, had a median value of 0.08% (range: 0–48.46%). At the TPS ≥1% threshold, 10 of 49 tumors (20.4%) were classified as PD-L1-positive, whereas 39 (79.6%) had a TPS <1%. The median CPS was 0 (IQR: 0–5; range: 0–90). No significant differences were found in immune cell populations and markers according to sex or *KIT* mutation type, and no expression of Arginase-1 was detected in the whole cohort. HLA-I complete or subclonal loss was found in 20.4% of GIST tumor samples (**Figures 2C–F**), supporting impaired antigen presentation as an alternative immune escape mechanism in at least a subset of GISTs. Concurrently, the induction of HLA-II expression in tumor cells was detected as a clonal event in a subset of GIST specimens.

**Figure 2:**
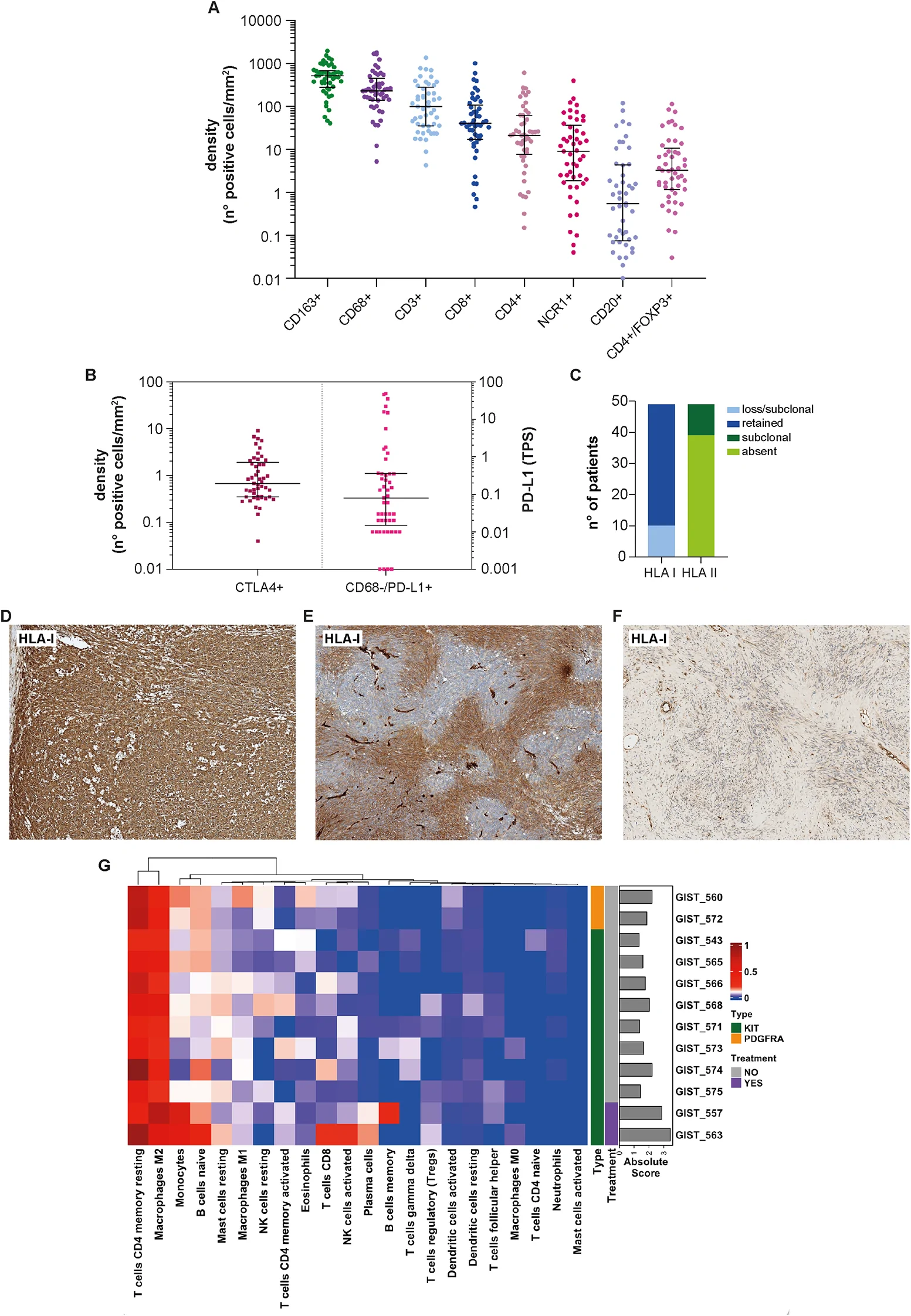
Immune cell portrait of the GIST tumor microenvironment. **(A)** Density of immune cell populations, expressed as positive cells per mm², across the analyzed GIST cohort. The panel includes markers for macrophages (CD68+, CD163+), T lymphocytes (CD3+, CD8+, CD4+), NK cells (NCR1+), B cells (CD20+), and regulatory T-cells (CD4+/FOXP3+). **(B)** Expression of immune checkpoints shown as density or tumor proportion score (TPS). PD-L1 TPS was calculated only for the tumor cell population (CD68-). Data are shown as individual points with median and interquartile range (IQR). **(C-F)**. HLA-I and HLA-II immunohistochemical expression patterns. **(C)** Proportion of patients showing HLA-I loss or subclonal loss and HLA-II subclonal positivity. **(D, F)** HLA-I immunohistochemical expression: representative images showing retained expression **(D)**, subclonal loss **(E)**, and loss of expression **(F)**. Magnification: 10x. **(G)** CIBERSORTx hierarchical clustering representing the abundance of 22 immune cell types (columns). The bar plot shows the absolute score summarizing all the immune population levels.

Bulk RNA sequencing was performed on 12 GIST samples, including 2 *PDGFRA*-mutant, 2 TKI-treated *KIT*-mutant, and 8 naive *KIT*-mutant tumors, and analyzed by CIBERSORTx deconvolution algorithm to characterize the immune cell composition of the TME (**Figure 2G**). Concordantly, the most represented immune populations were CD4+ T cells and M2 macrophages, followed by less abundant populations including B cells, NK cells and CD8+ T cells. Notably, tumor samples from patients previously treated with tyrosine kinase inhibitors showed higher CIBERSORTx absolute scores, with increased representation of selected immune subsets, including monocytes. Relative scores of the immune populations across all the samples can be found in **Supplementary Table 4**.

### HLA-I and HLA-II expression patterns are associated with immune cell infiltration

HLA-I expression was retained in 39 tumors (79.6%), while 8 (16.3%) showed subclonal loss and 2 (4.1%) complete loss. HLA-II expression was subclonal in 10 tumors (20.4%) and absent in 39 (79.6%). Tumors with subclonal or complete HLA-I loss showed lower densities of CD3+ and CD4+ T cells, CD4+/FOXP3+ regulatory T cells, and CD68+ macrophages (all *p* < 0.001), as well as CD163+ macrophages (*p* = 0.0023), NCR1+ cells (*p* = 0.0019), and CD8+ T cells (*p* = 0.028), compared with HLA-I-retained tumors. Similarly, HLA-II-negative tumors showed lower densities of CD3+ T cells (*p* = 0.005), CD4+/FOXP3+ regulatory T cells (*p* = 0.021), CD68+ macrophages (*p* = 0.014), and NCR1+ cells (*p* = 0.008) than tumors with subclonal HLA-II expression. HLA-I loss was also associated with lower PD-L1 TPS (*p* = 0.021), while HLA-II-negative tumors showed lower PD-L1 TPS and CPS (*p* = 0.0011 and *p* = 0.0002, respectively).

### Immune contexture by genotype, tumor site and disease presentation

Comparing the immune infiltrate between *PDGFRA*- and *KIT*-mutant primary GISTs revealed a distinct immunological profile in the former subgroup (**Figure 3**). Specifically, this cohort of patients exhibited higher levels of CD3+, CD20+ and NCR1+ immune populations, indicating increased infiltration of T lymphocytes, B cells, and NK cells, respectively. These differences were statistically significant when compared to *KIT*-mutant cases. Thus, GISTs harboring *PDGFRA* alterations show a more active immune microenvironment with enhanced lymphoid and innate immune cell infiltration. This pattern is consistent with the hypothesis that *PDGFRA*-associated mutational landscapes may be linked to increased tumor immunogenicity.

**Figure 3:**
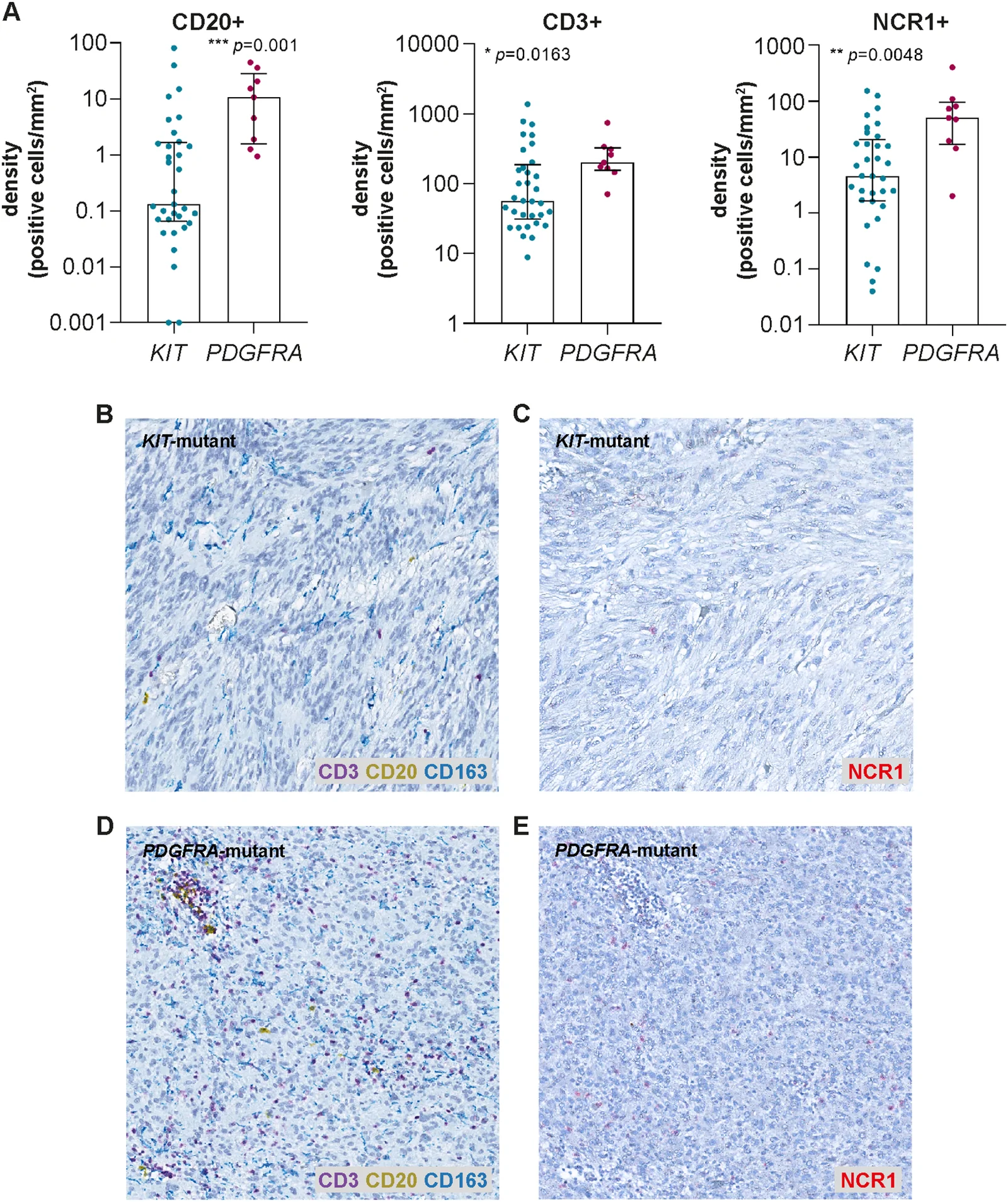
Comparison of immune marker expression between *KIT*- and *PDGFRA*-mutant primary GISTs. **(A)** Graphs representing the immune cell densities (expressed as positive cells per mm²) across *KIT*- and *PDGFRA*-mutated samples for CD20+, CD3+, NCR1 +. Data are presented as median with interquartile range (IQR). Y-axes are shown on a logarithmic scale. Statistical significance was determined by the Mann-Whitney test (*, *p<0.05*; **, p<0.01; ***, *p<0.001*). **(B–E)** Immunohistochemical expression of immune markers in *KIT*- and *PDGFRA*-mutant GIST. Representative images show CD3/CD20/CD163 triple staining **(B, D)** and CTLA-4/NCR1 double staining **(C, E)** in *KIT*-mutant GIST with low immune infiltration **(B, C)** and *PDGFRA*-mutant GIST with higher immune infiltration **(D, E)**. In the triple staining, CD3 is shown in purple, CD20 in yellow, and CD163 in teal; in the double staining, NCR1 is shown in red, while no CTLA-4+ cells are present in the representative fields. Magnification: 20×.

A tumor site-specific analysis of the immune infiltrate revealed quantitative differences between gastric and intestinal GISTs (**Figure 4**). Overall, intestinal tumors showed a higher degree of immune infiltration compared with gastric lesions. They were characterized by increased levels of CD3+, CD4+, and CD8+ T cells, indicating a greater representation of total T lymphocytes, T helper cells, and cytotoxic T cells. In addition, intestinal GISTs displayed higher numbers of CD4+/FOXP3+ regulatory T cells, as well as NCR1+ NK cells and CD68+ macrophages. Conversely, CD163+ M2 macrophages did not show any significant difference, suggesting that intestinal GISTs possibly display a less immunosuppressive macrophage infiltration. These findings indicate that intestinal GISTs are associated with a more prominent immune microenvironment compared with gastric tumors.

**Figure 4:**
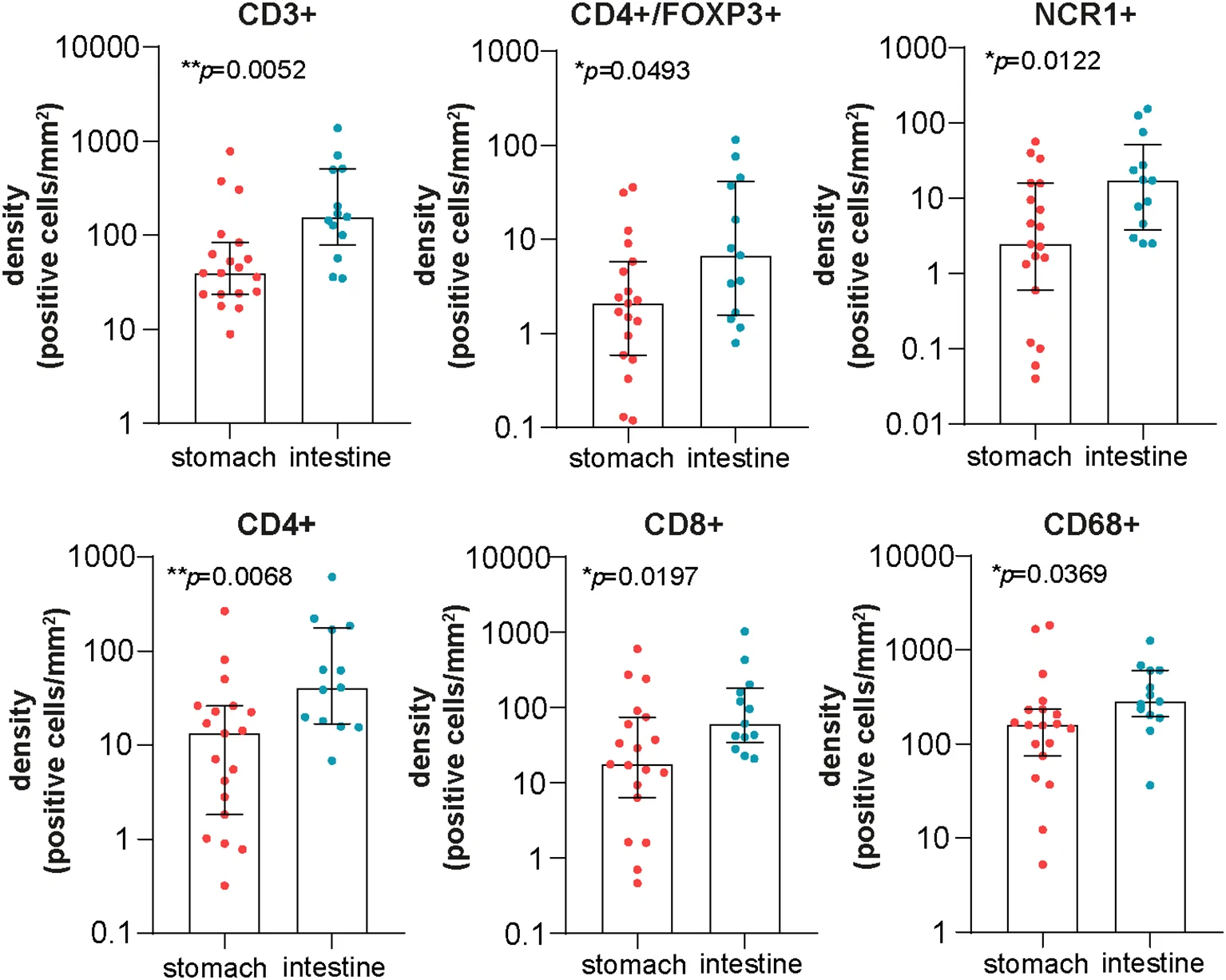
Comparison of immune cell populations based on tumor site in GIST patients. Graphs show immune cell densities (expressed as positive cells per mm²) in gastric and intestine (duodenum, jejunum, and ileum) GIST patients for the following markers: CD3+, CD4+/FOXP3+, NCR1+, CD4+, CD8+, CD68 +. Data are presented as median with interquartile range (IQR). Y-axes are shown on a logarithmic scale. Statistical significance was determined by the Mann-Whitney test (*, *p<0.05;* **, *p<0.01*).

Lastly, to assess whether metastatic progression is associated with alterations in the immune microenvironment, we compared the expression of immune-related markers in primary tumors and metastases from *KIT*-mutant GISTs (**Supplementary Figure 1**). No significant differences were observed for the analyzed markers. Notably, the comparable abundance of both innate and adaptive immune cell populations detected in primary and metastatic lesions reveals that metastatic *KIT*-mutant patients do not display a markedly altered immune landscape and may retain a comparable degree of immune recognition potential as observed in primary tumors.

Additional analysis of PD-L1 expression using the combined positive score (CPS) did not reveal statistically significant differences according to mutational genotype, tumor status, or anatomical site.

### TKI treatment reshapes the immune microenvironment in primary *KIT*-mutant tumors

To uncover the effect of TKI treatment on the tumor immune infiltrate, we compared the immune subpopulations in primary tumors between TKI-naive and TKI-treated patients (**Figure 5**). Exposure to imatinib was associated with a marked remodeling of the immune infiltrate in GIST samples. In particular, we observed a significant reduction in CD3+ T cell and NCR1+ NK cell density following treatment. This effect was even more pronounced for CD4+ T cells and regulatory T cells, identified as CD4+/FOXP3+, both of which showed a highly significant decrease in treated tumors. No significant difference was found in the CD8+ population, thus showing that the decrease in T-cells was driven mainly by Treg impairment. Imatinib treatment was also associated with a strong reduction in macrophage infiltration, as demonstrated by the decreased presence of both CD68+ and CD163+ cells. This finding supports a reduction in the immunosuppressive macrophage environment, particularly involving M2 macrophages. In contrast, no significant differences were observed in the abundance of CD20+ B cells between treated and naive samples. Overall, these findings reveal that imatinib profoundly affects the GIST immune microenvironment, mainly by reducing regulatory T-cell subsets, NK cells, and M2 macrophage populations.

**Figure 5:**
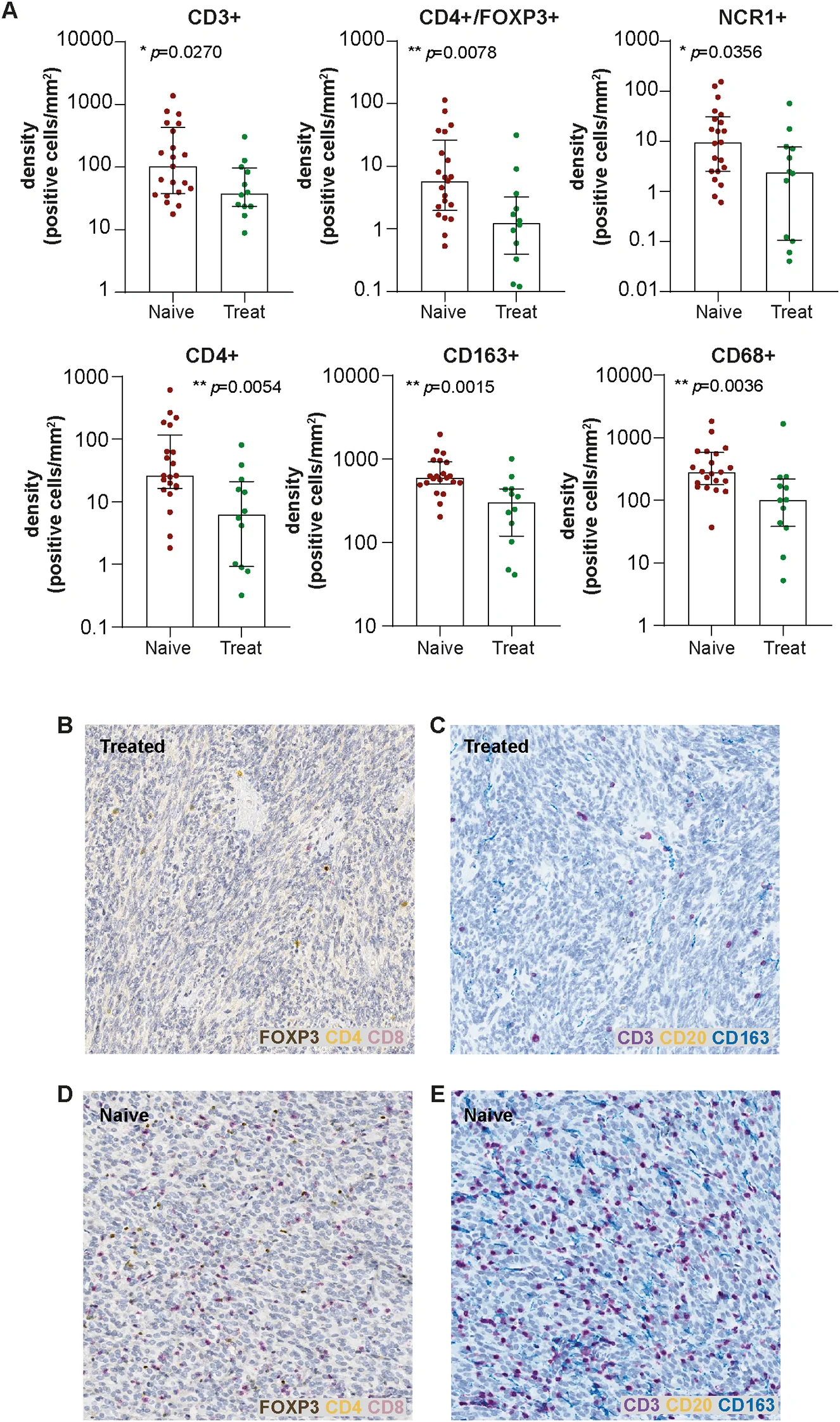
Comparison of immune populations in primary tumors between treated and treatment-naive GIST patients. **(A)** Graphs representing the immune cell densities (expressed as positive cells per mm²) across naive and treated (Treat) GIST patients’ primary tumors for CD3+, CD4+/FOXP3+, NCR1+, CD4+, CD163+, CD68 +. Data are presented as median with interquartile range (IQR). Y-axes are shown on a logarithmic scale. Statistical significance was determined by the Mann-Whitney test (*, *p<0.05*; **, p<0.01). **(B-E)** Immunohistochemical expression of immune markers in treated and treatment-naive GIST. Representative images show FOXP3/CD4/CD8 triple staining **(B, D)** and CD3/CD20/CD163 triple staining **(C, E)** in treated GIST with low immune infiltration **(B, C)** and treatment-naive GIST with higher immune infiltration **(D, E)**. In the FOXP3/CD8/CD4 stain, FOXP3 is shown in DAB, CD8 in purple, and CD4 in yellow; in the CD3/CD20/CD163 staining, CD3 is shown in purple, CD20 in yellow and CD163 in teal; no CD20+ cells are present in the representative fields. Magnification: 20×.

Additional analysis of PD-L1 expressing using CPS showed no statistically significant difference between TKI-treated and treatment-naive tumors.

## Discussion

The clinical efficacy of TKIs has changed the management of GISTs but remains limited overtime by the clonal selection of secondary resistance. Thus, the clinical need for a novel therapeutic approach remains urgent. The role of immunotherapy in GISTs is unclear since immune checkpoint inhibitors did not show impressive results as occurred in other solid tumors (17, 18). Therefore, investigating the immune microenvironment of this disease could help better understand this approach.

A comprehensive characterization of the tumor immune microenvironment of GIST samples depicted a rich and heterogeneous immune landscape. In this study, we identified an immune microenvironment with enrichment of macrophages, CD3+ T lymphocytes including CD8+, CD4+ and Treg populations and the significant presence of NK cells. Additionally, the levels of CD20 and CTLA-4 were consistently low. Overall, these findings highlight a complex immune landscape characterized by both innate and adaptive immune cell populations. Notably, this profile is consistent with the immune-rich microenvironment detected in our previous study, providing independent validation of those observations in this GIST cohort (10).

Moreover, the predominance of macrophages, and particularly of CD163+ M2-like macrophages, confirms that myeloid cells represent a major component of the GIST immune microenvironment (9, 12, 30, 31). This observation suggests that immune infiltration in GISTs is not necessarily equivalent to effective antitumor immunity, since a relevant fraction of the immune infiltrate may be represented by immunosuppressive macrophage populations. The recent demonstration that GIST cells may promote macrophage M2 polarization through the MIF/CXCR4 axis further supports the role of the macrophage-driven immune escape in this disease (31). Notably, one of the novel findings of our study is the identification of complete or subclonal HLA-I loss in a subset of tumors. Previous studies have mainly focused on the immune-cell composition, immune signatures, checkpoint molecules, and macrophage polarization (9–11, 31), while the antigen-presentation machinery has been less systematically investigated. Importantly, our findings also revealed a relationship between HLA expression and the composition of the immune microenvironment. Tumors with subclonal or complete HLA-I loss showed lower infiltration by multiple immune-cell populations, including CD3+, CD4+, and CD8+ T cells, CD4+/FOXP3+ regulatory T cells, CD68+ and CD163+ macrophages, and NCR1+ cells. A similar pattern was observed for HLA-II, as tumors lacking HLA-II expression showed lower densities of CD3+, CD4+/FOXP3+, CD68+, and NCR1+ cells than tumors with subclonal HLA-II expression. HLA-I loss was also associated with lower PD-L1 TPS, while HLA-II-negative tumors showed lower PD-L1 TPS and CPS. Taken together, these findings indicate that reduced HLA expression is associated with a less immune-infiltrated tumor microenvironment in GIST, linking antigen-presentation status to the broader immune contexture of these tumors. However, the observational nature of our study does not establish whether HLA loss is a cause or a consequence of reduced immune infiltration, nor does it directly demonstrate immune escape. Functional studies will therefore be required to determine the biological consequences of HLA loss and its potential relevance to response or resistance to immunotherapy.

To better understand how the immune landscape is associated with GIST clinical heterogeneity, we correlated it with clinical and pathological characteristics.

The 9 GISTs harboring *PDGFRA* alterations showed an enhanced lymphoid and innate immune cell infiltration compared with GISTs harboring *KIT* mutations (15). These data are consistent with those provided in previous studies and are now confirmed in a larger series with a more comprehensive approach (9, 10, 32). Importantly, our data extended observations at the tissue level by showing that the immune-enriched profile of *PDGFRA*-mutant GISTs includes not only T lymphocytes, but also B- and NK cells. The enrichment of CD20+ B cells is particularly noteworthy, since B cells have been less extensively investigated than T cells and macrophages in GIST immune profiling. This finding raises the possibility that humoral immune mechanisms, antigen presentation by B cells, or tertiary lymphoid-like immune organization may contribute to the immunological identity of *PDGFRA*-mutant GISTs. Actually, the B-cell enrichment observed in *PDGFRA*-mutated GISTs may suggest the presence of tertiary lymphoid structures, although histological and spatial validation is required.

Moreover, since patients with *PDGFRA* mutations show a more favorable natural history when compared to those with *KIT* mutations, this pattern is consistent with the hypothesis that *PDGFRA*-associated mutational landscapes may be linked to increased tumor immunogenicity.

Vitiello and colleagues studied 75 GISTs, of which 24 had *PDGFRA* mutations, by performing RNA-sequencing, immunohistochemistry and flow cytometry, confirming that *PDGFRA*-mutant GISTs appear to be more immunologically active than *KIT*-mutant GISTs (9). They hypothesized that the mutational driver may impact other aspects of tumor biology, specifically the TME and host immune response. Additionally, considering the known heterogeneity within *PDGFRA*-mutant GISTs, in our previous experience a subset of 10 samples of primary gastric *PDGFRA*-mutant GIST, including 5 cases with the D842V mutation and 5 with mutations other than D842V, was analyzed (15). The D842V-mutant cases presented a significant enrichment of immune- and interferon-related gene signatures. Gene expression analysis revealed a significantly higher abundance of CD8+ T-cells, while transcriptomic profiling identified the presence of the T cell-inflamed signature (TIS) in D842V patients. Moreover, the D842V-mutant GISTs presented a peculiar downregulation of several oncogenes, transcription factors, and nuclear receptors. This feature, along with the confirmed immunogenicity, may be associated with the characteristic indolent course of the D842V-mutant subgroup. Interestingly, our findings are consistent with this hypothesis and suggest that the more favorable behavior of *PDGFRA*-mutant GISTs may be related not only to intrinsic oncogenic signaling, but also to a more permissive immune contexture. However, whether this immune-enriched phenotype directly contributes to the indolent clinical course or simply reflects the distinct biology of *PDGFRA*-mutant tumors remains to be clarified in larger cohorts with clinical outcome correlations.

Tumor site also emerged as a relevant determinant of immune contexture in our cohort. Intestinal GISTs showed higher levels of CD3+, CD4+, CD8+, CD4+/FOXP3+, NCR1+, and CD68+ cells compared with gastric tumors, whereas CD163-positive M2-like macrophages did not significantly differ. This finding agrees with the concept that tumor location contributes to GIST immunogenicity, as demonstrated by Gasparotto and colleagues, who showed that tumor genotype, location, and malignant potential shape the immunogenicity of primary untreated GISTs (11). In our series, the increased immune infiltration observed in intestinal GISTs did not appear to be accompanied by a parallel increase in M2-like macrophages, suggesting a quantitatively richer and potentially more immune-active microenvironment rather than a merely more suppressive macrophage-dominated phenotype.

To assess the association with tumor progression, we evaluated the expression of immune-related markers in primary tumors and metastatic lesions from *KIT*-mutant cases, finding no difference in the abundance of both innate and adaptive immune cell populations. These findings suggest that the overall immune composition remains largely stable during metastatic progression. Remarkably, this suggests that metastatic progression in *KIT*-mutant GISTs is not necessarily associated with broad immune exclusion or a simple quantitative reduction of immune-cell infiltration. Specifically, metastatic disease may retain a comparable degree of immune recognition potential, at least in terms of immune-cell abundance. This observation may help refine current models of GIST progression, indicating that immune escape during metastatic dissemination could be mediated by qualitative rather than quantitative changes in the tumor immune microenvironment.

Recently, Li and colleagues investigated alterations in the immune microenvironment during GIST progression and found that MYBL1 and AIF1L could be key mediators regulating the activation of CD8+ T cell function and the suppression of PD‐1 response respectively, providing the basis for enhancing the efficacy of PD‐1 blockade as a new strategy for combined immunotherapy in GISTs compared to current treatments (33). In our cohort, we did not observe significant differences in the abundance of innate and adaptive immune-cell populations between primary and metastatic *KIT*-mutant GISTs. This apparent discrepancy may be explained by the different biological layers captured by the two approaches. While our analysis mainly assessed the quantitative composition of selected immune-cell subsets, multi-omic and single-cell approaches can reveal functional, transcriptional, and cell–cell communication changes that may occur without major variations in immune-cell density. Thus, metastatic progression in GISTs may not require broad immune exclusion, but may depend on qualitative immune remodeling, including impaired CD8+ T-cell activity, altered PD-1-related pathways, macrophage polarization, defective antigen presentation, or spatial reorganization of immune cells.

However, understanding the dynamic immune characteristics in advanced GISTs remains challenging since most available data regarding metastatic samples present confounding factors such as the TKI therapy and the consequent impact on the tumor microenvironment.

Indeed, treatment exposure represents one of the major confounders in the interpretation of the immune landscape of advanced GISTs. In our case series, we decided to evaluate the effect of TKI in primary tumors, to minimize the potential confounding effects. TKI-treated primary *KIT*-mutant tumors showed a significant reduction in CD3+ T cells, CD4+ cells, CD4+/FOXP3+ regulatory T cells, NCR1+ NK cells, CD68+ macrophages, and CD163+ macrophages, whereas CD8+ T cells and B cells were relatively preserved. These data indicate that imatinib does not simply induce global immune depletion but rather remodels the immune microenvironment by preferentially reducing suppressive or regulatory compartments, including Tregs and M2-like macrophages. Interestingly, the two TKI-treated cases included in the CIBERSORTx analysis displayed the highest immune scores. Although this finding may appear to be in contrast with the immunohistochemistry results, it should be interpreted with caution, given the very limited number of analyzed samples compared with the IHC cohort. Nevertheless, it may provide an additional indication of immune microenvironment remodeling in TKI-treated cases, highlighting the complexity and heterogeneity of the immune changes associated with treatment history.

Balachandran and colleagues also demonstrated that imatinib potentiates antitumor T-cell responses in GISTs through the inhibition of IDO and reduction of immunosuppressive mechanisms (16). This is in line with the preclinical evidence provided by Zhang and colleagues, showing that macrophages and CD8+ T cells mediate the antitumor efficacy of combined CD40 ligation and imatinib therapy in GISTs (13). Although these studies provide evidence that TKI treatment may influence the immune microenvironment, the reduction of CD163+ macrophages and Tregs observed in our treated cases is accompanied by a concomitant decrease in other immune populations. Therefore, our data suggest a broader remodeling of immune-cell infiltration rather than a selective depletion of immunosuppressive populations. Although these findings are consistent with an immunomodulatory effect of TKI treatment, they do not provide sufficient evidence to establish the specific mechanisms described in previous experimental studies.Currently, clinical experiences in GIST patients treated with immunotherapy alone or combined with TKIs with anti-angiogenic effects are available. These studies with a small number of patients portrayed limited antitumor activity in terms of response rate and progression free survival (PFS) (17, 34). The Axagist trial evaluating axitinib in combination with avelumab in GIST patients described a response and stable disease in more than 60% of cases, while among patients exhibiting partial response, the median duration of response was 18.5 months (18). The median PFS and overall survival were 4.6 months and 14.2 months respectively. Even though this combination resulted in long-term clinical benefits in a significant subset of patients, the immune checkpoint inhibition in GISTs has not delivered the same transformative impact seen in other solid tumors. Consequently, the authors concluded that further research studies are needed to understand the mechanisms of resistance to immunotherapy and to determine which subgroup of patients should be selected for future studies.

Overall, the results of our study, together with the clinical findings, support a model in which GIST immune biology is shaped by multiple interacting factors: oncogenic genotype, anatomical site, treatment exposure, macrophage polarization, checkpoint activation, and antigen-presentation competence. This comprehensive view may help explain the limited efficacy of immunotherapy in unselected GIST patients and support the development of biomarker-driven combination strategies that account for molecular subtype, tumor location, TKI-induced immune remodeling and HLA-I status.

These observations have several translational implications. First, the predominance of macrophages and the modulation of CD163+ cells after imatinib support macrophage-targeted or macrophage-reprogramming strategies as rational therapeutic approaches. Second, the relative preservation of CD8+ T cells after TKI exposure suggests that immunotherapeutic combinations may be more effective when applied in a context characterized by reduced suppressive immune compartments and retained cytotoxic effectors. Third, the heterogeneous expression of PD-L1 and CTLA-4, together with the limited activity of checkpoint-based clinical strategies in refractory GISTs, indicates that the immune checkpoint inhibition should probably be explored in biomarker-selected populations rather than in unselected patients (18, 34, 35). Finally, the identification of HLA-I loss in a subset of tumors suggests that the antigen-presentation status should be incorporated into future immunotherapy trials and biomarker studies. Indeed, HLA-I loss may represent an immune-evasion mechanism by impairing CD8+ T-cell recognition and potentially reducing the efficacy of T-cell-based immunotherapies. Conversely, it may increase susceptibility to NK-cell-mediated killing, supporting the investigation of NK-cell-based or other HLA-independent therapeutic strategies. Indeed, based on the immune escape mechanisms identified in this study, we can envisage potential approaches deserving preclinical investigation that include combining TKIs with CD40 agonists or other macrophage-directed agents, cytokine- or checkpoint-based NK-cell activation, and, in tumors showing reversible HLA-I downregulation, interferon- or epigenetic-based restoration of antigen presentation. Among these strategies, CD40 agonism combined with imatinib has already shown antitumor activity in preclinical GIST models (13).

Finally, future studies should characterize the functional status of CD8^+^ T cells after imatinib treatment using multiplex analysis of inhibitory, activation and cytotoxic markers, including PD-1, LAG-3, Ki-67 and granzyme B. Future perspective studies integrating standardized clinical outcome data with functional immune assays, including tumor-specific T-cell cytotoxicity and NK-cell activity, will be required to determine the clinical and biological consequences of the immune features identified in this study.

Several limitations should also be acknowledged, starting with the retrospective design and the relatively small sample size which is mainly attributable to the rarity of these tumors, especially those harboring *PDGFRA* alterations. These factors may reduce the generalizability of the results and the statistical power of subgroup analysis. This limitation, together with the poor quality of certain FFPE samples, further reduced the number of cases available for RNA sequencing, enhancing the risk of selection bias and limiting the robustness of the transcriptomic findings. Therefore, the subgroup and transcriptomic analysis should be considered exploratory. Secondly, the functional consequences of HLA class I loss remain to be demonstrated. Although our findings are consistent with a potential role of HLA-I loss in immune evasion and therapy resistance, no mechanistic or clinical outcome data were available to establish a direct association between HLA-I loss and these outcomes. Furthermore, the cross-sectional nature of our analysis does not allow the assessment of dynamic changes in the immune microenvironment over time or in response to therapy. Another drawback is represented by the lack of tumor mutational burden (TMB) data since the TMB may influence tumor biology and immune-related features. Thus, we could not assess its potential contribution to the molecular profiles in our cohort. Despite these limitations and the limited statistical power of some analysis, our results are often supported by the existing literature and pave the way for more in-depth analysis, including longitudinal studies and validation in larger, independent cohorts.

In conclusion, our study confirms that GISTs harbor a rich and heterogeneous immune microenvironment and provides new evidence that this landscape is shaped by genotype, tumor site, TKI exposure, and antigen-presentation status. Therefore, a therapeutic approach with immunological modulators along with TKIs in the treatment of GIST should consider the effects of all the variables previously mentioned, which would be assessed in terms of both additive and interactive effects. The main findings include the association of HLA-I loss and absent HLA-II expression with reduced immune cell infiltration as a potential immune escape mechanism, the demonstration of a more lymphoid-, B-cell-, and NK-enriched immune profile in *PDGFRA*-mutant tumors, the site-specific immune enrichment of intestinal GISTs, and the selective remodeling of regulatory and myeloid immune compartments after imatinib treatment. These results refine the current understanding of GIST immunobiology and support the development of rational, biomarker-driven immunotherapeutic strategies.

## Data Availability

The original contributions presented in the study are publicly available. This data can be found here: https://www.ncbi.nlm.nih.gov/, accession number: PRJNA1531162.
