# Supplementary material for: Mapping the immune microenvironment of KIT- and PDGFRA-mutant GISTs reveals genotype-specific immune infiltration and TKI-induced remodeling
Source: Front Immunol. 2026 Oct 15;17:1961524. doi: 10.3389/fimmu.2026.1961524 (PMC13717502; doi:10.3389/fimmu.2026.1961524)
Supplement: Supplementary file 1 [file Table1.docx]

**Supplemental materials**

**Supplementary Table 1.** GIST tumor and clinical characteristics of patients.

| **Patient ID** | **Sex** | **Age at diagnoses** | **Stage at diagnoses** | **Primary tumor site** | **Metastatic site** | **Tumor size (mm)** | **Mitotic Index** | **Risk stratification*** | **KIT/PDGFRα mutation** | **Exon** | **Tumor Sample** | **Pre-treated** | **Drug** |
| --- | --- | --- | --- | --- | --- | --- | --- | --- | --- | --- | --- | --- | --- |
| 1 | M | 62 | localized | stomach |  | 70 | NA | High | KIT | 11 | Primary tumor | yes | IM |
| 2 | M | 43 | localized | duodenum |  | 70 | NA | High | KIT | 11 | Primary tumor | yes | IM |
| 3 | F | 34 | localized | stomach |  | 65 | NA | High | KIT | 9 | Primary tumor | yes | IM |
| 4 | F | 67 | advanced | stomach | liver | 80 | >5/50HPF | NA | KIT | 11 | Primary tumor | no | IM |
| 5 | M | 65 | localized | stomach |  | 180 | NA | High | KIT | 11 | Metastatic lesion | yes | IM |
| 6 | F | 65 | localized | stomach |  | 180 | >5/50HPF | High | KIT | 11 | Primary tumor | no | IM |
| 7 | M | 58 | localized | ileum |  | 60 | 3/50HPF | Intermedium | KIT | 11 | Metastatic lesion | yes | SU |
| 8 | M | 76 | localized | stomach |  | 45 | 6/50HPF | Intermedium | PDGFRα | 18 | Primary tumor | no | IM |
| 9 | M | 70 | localized | stomach |  | 170 | >10/50HPF | High | KIT | 11 | Metastatic lesion | yes | IM |
| 10 | M | 68 | localized | stomach |  | 30 | 5/50HPF | Very low | PDGFRα | 18 | Primary tumor | no | IM |
| 11 | F | 45 | localized | stomach |  | 18 | 3/50HPF | Very low | PDGFRα | 18 | Primary tumor | no | IM |
| 12 | F | 77 | localized | stomach |  | 200 | NA | High | KIT | 11 | Primary tumor | yes | IM |
| 13 | F | 75 | localized | stomach |  | 70 | 8/50HPF | High | PDGFRα | 18 | Primary tumor | no | IM |
| 14 | M | 35 | localized | ileum |  | 120 | 64/50HPF | High | KIT | 11 | Primary tumor | no | IM |
| 15 | F | 56 | advanced | stomach | liver | 70 | 68/50HPF | NA | KIT | 11 | Primary tumor | no | IM |
| 16 | M | 51 | localized | stomach |  | 120 | <2/50HPF | Intermedium | PDGFRα | 18 | Primary tumor | no | IM |
| 17 | F | 58 | localized | stomach |  | 100 | 14/50HPF | NE | KIT | 11 | Primary tumor | yes | IM |
| 18 | M | 59 | advanced | stomach | liver | 100 | NA | NA | KIT | 11 | Primary tumor | yes | IM |
| 19 | M | 59 | localized | stomach |  | 120 | NA | NE | KIT | 9 | Primary tumor | yes | IM |
| 20 | M | 50 | localized | stomach |  | 80 | 2/50HPF | Low | KIT | 11 | Metastatic lesion | yes | SU |
| 21 | M | 51 | localized | stomach |  | 70 | 5/50HPF | Low | PDGFRα | 14 | Primary tumor | no | IM |
| 22 | F | 55 | localized | stomach |  | 75 | 6/50HPF | High | KIT | 11 | Primary tumor | no | IM |
| 23 | F | 41 | localized | stomach |  | 70 | 30/50HPF | High | KIT | 11 | Metastatic lesion | yes | SU |
| 24 | F | 58 | localized | ileum |  | NA (capsule rupture) | 10/50HPF | High | KIT | 11 | Metastatic lesion | yes | IM |
| 25 | F | 66 | localized | ileum |  | 24 | 9/50HPF | High | KIT | 9 | Primary tumor | no | IM |
| 26 | M | 34 | localized | stomach |  | 27 | 4/50HPF | Very low | KIT | 11 | Primary tumor | no | IM |
| 27 | F | 59 | advanced | stomach | liver | 30 | NA | NA | KIT | 11 | Metastatic lesion | no | IM |
| 28 | M | 79 | localized | stomach |  | 40 | 6/50HPF | Intermedium | KIT | 11 | Primary tumor | no | IM |
| 29 | F | 48 | localized | stomach |  | 85 | 5/50HPF | Low | KIT | 9 | Primary tumor | no | IM |
| 30 | M | 46 | localized | stomach |  | 20 | 6/50HPF | Null | KIT | 11 | Primary tumor | no | IM |
| 31 | F | 72 | localized | stomach |  | 32 | NA | Low | KIT | 11 | Primary tumor | yes | IM |
| 32 | F | 40 | localized | ileum |  | 150 | NA | High | KIT | 11 | Primary tumor | yes | IM |
| 33 | M | 54 | localized | jejunum |  | 70 | 4/50HPF | Intermedium | KIT | 11 | Primary tumor | no | IM |
| 34 | F | 85 | localized | stomach |  | 40 | 4/50HPF | Very low | PDGFRα | 14 | Primary tumor | no | IM |
| 35 | M | 49 | localized | ileum |  | 12 | 2/50HPF | Null | KIT | 11 | Primary tumor | no | IM |
| 36 | F | 46 | localized | stomach |  | 110 | NA | NE | KIT | 11 | Primary tumor | yes | IM |
| 37 | M | 68 | localized | stomach |  | 105 | NA | NE | KIT | 11 | Primary tumor | yes | IM |
| 38 | M | 65 | localized | jejunum |  | 30 | 3/50HPF | Low | KIT | 11 | Primary tumor | no | IM |
| 39 | F | 58 | localized | colon |  | 65 | 7/50HPF | High | KIT | 11 | Primary tumor | no | IM |
| 40 | F | 63 | advanced | ileum | peritoneum | 90 | >10/50HPF | NA | KIT | 9 | Primary tumor | no | IM |
| 41 | F | 48 | localized | stomach |  | 110 | 4/50HPF | Intermedium | PDGFRα | 18 | Primary tumor | no | IM |
| 42 | F | 68 | localized | stomach |  | 25 | <5/50HPF | Very low | KIT | 11 | Primary tumor | no | IM |
| 43 | F | 73 | advanced | stomach | liver | 140 | NA | NA | KIT | 11 | Primary tumor | yes | IM |
| 44 | M | 73 | localized | ileum |  | 35 | 4/50HPF | Very low | KIT | 13 | Primary tumor | no | IM |
| 45 | F | 53 | localized | ileum |  | 35 | 3/50HPF | Low | KIT | 9 | Primary tumor | no | IM |
| 46 | M | 64 | localized | stomach |  | 7 | 2/50HPF | Null | PDGFRα | 18 | Primary tumor | no | IM |
| 47 | F | 62 | localized | jejunum |  | 38 | 6/50HPF | High | KIT | 11 | Primary tumor | no | IM |
| 48 | F | 50 | localized | jejunum |  | 85 | 3/50HPF | Intermedium | KIT | 11 | Primary tumor | no | IM |
| 49 | M | 68 | localized | duodenum |  | 32 | 5/50HPF | Low | KIT | 9 | Primary tumor | no | IM |

Abbreviations: F = female; HPF = high power field; IM = imatinib; M = male; NE not evaluable; NA = not applicable; PDGFRA = platelet-derived growth factor receptor α; SU = sunitinib.

*According to risk-stratification criteria by Miettinen and Lasota (35).

**Supplementary Table 2A. Antibodies specifications.** Abbreviations: RTU = Ready to use

| **Marker** | **Specie** | **Clone** | **Dilution** | **Source** |
| --- | --- | --- | --- | --- |
| Arginase-1 | Rabbit | SP156 | RTU | Cell Marque, USA |
| HLA-I | Mouse | EMR8-5 | 1:2200 | Abcam, UK |
| HLA-II | Mouse | LGII-612.14 | 1:6000 | Cell Signaling, USA |
| CTLA-4 | Mouse | BSB-88 | 1:200 | Bio SB, USA |
| NCR1 | Rabbit | EPR22403-57 | 1:250 | Abcam, UK |
| PD-L1 | Rabbit | SP263 | RTU | Ventana, USA |
| CD68 | Mouse | PG-M1 | RTU | Diagnostic Biosystems, USA |
| CD3 | Rabbit | 2GV6 | RTU | Ventana, USA |
| CD20 | Mouse | L26 | RTU | Ventana, USA |
| CD163 | Mouse | MRQ-26 | RTU | Cell Marque, USA |
| FOXP3 | Mouse | 236A/E7 | 1:200 | Abcam, UK |
| CD8 | Rabbit | SP57 | RTU | Ventana, USA |
| CD4 | Rabbit | SP35 | RTU | Ventana, USA |

**Supplementary Table 2B.** Immunohistochemical protocols for single, dual, and triple-marker staining.

| **Staining** | **Markers** | **Antigen retrieval** | **Incubation** | **Visualization** |
| --- | --- | --- | --- | --- |
| Single IHC | Arginase-1 | Ultra CC1 95°C x 32 min | 37°C x 16 min | DAB |
| Single IHC | HLA-I | Ultra CC1 95°C x 24 min | 36°C x 20 min | DAB |
| Single IHC | HLA-II | Ultra CC1 95°C x 32 min | 36°C x 20 min | DAB |
| Dual IHC | CTLA-4  NCR1 | Ultra CC1 95°C x 32 min  - | RT x 28 min  RT x 32 min | DAB + Amp  Red + Amp |
| Dual IHC | PD-L1  CD68 | Ultra CC1 99°C x 64 min  - | 36°C x 12 min  36°C x 24 min | DAB  Red |
| Triple IHC | CD3  CD20  CD163 | Ultra CC1 95°C x 24 min  Ultra CC1 95°C x 8 min  - | 36°C x 20 min  36°C x 20 min  36°C x 20 min | Purple  Yellow  Teal |
| Triple IHC | FOXP3  CD8  CD4 | Ultra CC1 95°C x 56 min  -  - | 36°C x 32 min  36°C x 20 min  36°C x 32 min | DAB  Purple  Yellow |

Abbreviations: IHC = Immunohistochemistry; Ultra CC1 = Ultra Cell Conditioning; RT = Room temperature; DAB = OptiView DAB Detection Kit; Red = ultraView Universal Alkaline Phosphatase Red Detection Kit; Amp = Amplification kit; Purple = DISCOVERY Purple Kit; Yellow = DISCOVERY Yellow HRP Kit; Teal = DISCOVERY Teal HRP Kit. All reagents were from Ventana Medical Systems/Roche Diagnostics, Tucson, AZ, USA.

**Supplementary Figure 1. Immune landscape of primary and metastatic *KIT*-mutated GISTs.** Graphs represent the immune cell densities (expressed as percentage of positive cells per mm²) across primary and metastatic *KIT*-mutated samples for CD20+, CD3+, CD4+/FOXP3+, CD4+, CD8+, NCR1+, CD163+, CD68+, and CTLA-4+. CD68-/PD-L1+ expression is reported as a percentage of positive tumor cells. Data are presented as medians with interquartile ranges (IQRs) and were analyzed using the Mann-Whitney test, except for CD163+, which is presented as mean with standard deviation (SD) and was analyzed using Welch’s unpaired *t*-test. Y-axes are shown on a logarithmic scale. (Primitive, primary tumor; Mets, metastases).

**Supplementary Table 3.** Descriptive statistics, normality assessment, between-group difference estimates, and 95% confidence intervals for all evaluated immunological markers.

| **Figure** | **Marker** | **Gr. 1** | **Gr2** | **n*1*** | **n2** | **Shapiro-Wilk *p*, Gr. 1** | **Shapiro-Wilk *p*, Gr. 2** | **Hodges-Lehmann** | **Mean difference** | **95% CI** | **Mann-Whitney U** | **Welch’s t (df)** | **Exact**  ***p*-value** | **FDR adjusted *q-*value** |
| --- | --- | --- | --- | --- | --- | --- | --- | --- | --- | --- | --- | --- | --- | --- |
| Fig. 3 | CD20+ | *KIT* | *PDGFRA* | 33 | 9 | <0.0001 | 0.0830 | 6.430 | NA | 1.160, 9.03 | 46 | NA | 0.0010 | 0.0090 |
| Fig. 3 | CD3+ | *KIT* | *PDGFRA* | 33 | 9 | <0.0001 | 0.0165 | 128.9 | NA | 31.32, 211.7 | 71 | NA | 0.0163 | 0.0489 |
| Fig. 3 | NCR1+ | *KIT* | *PDGFRA* | 33 | 9 | <0.0001 | 0.0005 | 44.61 | NA | 11.35, 72.69 | 59 | NA | 0.0048 | 0.0216 |
| NDF | CD8+ | *KIT* | *PDGFRA* | 33 | 9 | <0.0001 | 0.0007 | 14.49 | NA | -28.71, 46.04 | 117 | NA | 0.3482 | 0.4477 |
| NDF | CD163+ | *KIT* | *PDGFRA* | 33 | 9 | 0.0021 | 0.312 | 41.78 | NA | -262.5, 384.1 | 140 | NA | 0.8096 | 0.8096 |
| NDF | CD4+/  FOXP3+ | *KIT* | *PDGFRA* | 33 | 9 | <0.0001 | <0.0001 | 0.730 | NA | -3.530, 5.910 | 132 | NA | 0.6295 | 0.7082 |
| NDF | CD4+ | *KIT* | *PDGFRA* | 33 | 9 | <0.0001 | 0.0023 | 7.830 | NA | -12.70, 40.32 | 116 | NA | 0.3327 | 0.4477 |
| NDF | CTLA4+ | *KIT* | *PDGFRA* | 33 | 9 | <0.0001 | <0.0001 | -0.220 | NA | -0.930, 0.090 | 104 | NA | 0.1780 | 0.3204 |
| NDF | CD68-/  PD-L1+ | *KIT* | *PDGFRA* | 33 | 9 | <0.0001 | <0.0001 | 0.270 | NA | -0.010, 1.500 | 97.50 | NA | 0.1172 | 0.2637 |
| Fig. 4 | CD3+ | stomach | intestine | 19 | 13 | <0.0001 | 0.0011 | 106.5 | NA | 25.98, 186.8 | 52 | NA | 0.0052 | 0.0340 |
| Fig. 4 | CD4+/  FOXP3+ | stomach | intestine | 19 | 13 | <0.0001 | 0.0009 | 3.280 | NA | 0.08, 16.2 | 72 | NA | 0.0493 | 0.0822 |
| Fig. 4 | NCR1+ | stomach | intestine | 19 | 13 | <0.0001 | 0.0006 | 7.700 | NA | 1.180, 22.84 | 59 | NA | 0.0122 | 0.0407 |
| Fig. 4 | CD4+ | stomach | intestine | 19 | 13 | <0.0001 | 0.0002 | 27.72 | NA | 8.400, 88.28 | 54 | NA | 0.0068 | 0.0340 |
| Fig. 4 | CD8+ | stomach | intestine | 19 | 13 | <0.0001 | <0.0001 | 33.74 | NA | 6.100, 107.1 | 63 | NA | 0.0197 | 0.0493 |
| Fig. 4 | CD68+ | stomach | intestine | 19 | 13 | <0.0001 | 0.0253 | 128.4 | NA | 24.16, 299.1 | 69 | NA | 0.0369 | 0.0738 |
| NDF | CD20+ | stomach | intestine | 19 | 13 | <0.0001 | <0.0001 | 0.290 | NA | -0.300, 1.670 | 82.50 | NA | 0.1188 | 0.1697 |
| NDF | CD163+ | stomach | intestine | 19 | 13 | 0.0012 | 0.6897 | 128.9 | NA | -90.81, 342.2 | 93 | NA | 0.2538 | 0.2820 |
| NDF | CTLA4+ | stomach | intestine | 19 | 13 | <0.0001 | 0.0004 | -0.180 | NA | -0.980, 0.340 | 106 | NA | 0.5134 | 0.5134 |
| NDF | CD68-/  PD-L1+ | stomach | intestine | 19 | 13 | <0.0001 | <0.0001 | 0.020 | NA | -0.020, 0.240 | 91 | NA | 0.2167 | 0.2709 |
| Fig. 5 | CD3+ | naive | treated | 21 | 12 | <0.0001 | 0.0009 | -40.94 | NA | -180.1, -3.100 | 67 | NA | 0.0270 | 0.0540 |
| Fig. 5 | CD4+/  FOXP3+ | naive | treated | 21 | 12 | <0.0001 | <0.0001 | -3.285 | NA | -11.26, -0.670 | 56 | NA | 0.0078 | 0.0195 |
| Fig. 5 | NCR1+ | naive | treated | 21 | 12 | <0.0001 | <0.0001 | -4.530 | NA | -19.04, -0.240 | 70 | NA | 0.0356 | 0.0593 |
| Fig. 5 | CD4+ | naive | treated | 21 | 12 | <0.0001 | 0.0009 | -19.13 | NA | -55.17, -5.570 | 53 | NA | 0.0054 | 0.0180 |
| Fig. 5 | CD163+ | naive | treated | 21 | 12 | 0.0024 | 0.1243 | -327.1 | NA | -527.6, -148.1 | 44 | NA | 0.0015 | 0.0150 |
| Fig. 5 | CD68+ | naive | treated | 21 | 12 | <0.0001 | <0.0001 | -164.3 | NA | -320.7, -61.99 | 50 | NA | 0.0036 | 0.0180 |
| NDF | CD20+ | naive | treated | 21 | 12 | <0.0001 | 0.0001 | -0.050 | NA | -0.690, 0.860 | 101 | NA | 0.3596 | 0.3596 |
| NDF | CD8+ | naive | treated | 21 | 12 | <0.0001 | 0.0043 | -15.93 | NA | -59.32, 22.28 | 94 | NA | 0.2424 | 0.2704 |
| NDF | CTLA4+ | naive | treated | 21 | 12 | 0.0026 | <0.0001 | -0.610 | NA | -1.330, 0.140 | 83.50 | NA | 0.1147 | 0.1639 |
| NDF | CD68-/  PD-L1+ | naive | treated | 21 | 12 | <0.0001 | <0.0001 | 0.019 | NA | -0.020, 0.150 | 94.50 | NA | 0.2434 | 0.2704 |
| Fig. S1 | CD20+ | primitive | mets | 12 | 6 | 0.0001 | <0.0001 | 0.150 | NA | -1.110, 0.480 | 33 | NA | 0.8007 | 0.9999 |
| Fig. S1 | CD3+ | primitive | mets | 12 | 6 | 0.0009 | 0.1026 | 64.68 | NA | -29.13, 334.4 | 25 | NA | 0.3355 | 0.9999 |
| Fig. S1 | CD4+/  FOXP3+ | primitive | mets | 12 | 6 | <0.0001 | 0.0067 | 0.315 | NA | -1.600, 13.12 | 32.50 | NA | 0.7671 | 0.9999 |
| Fig. S1 | NCR1+ | primitive | mets | 12 | 6 | <0.0001 | 0.0057 | 0.730 | NA | -5.390, 13.29 | 28 | NA | 0.4936 | 0.9999 |
| Fig. S1 | CD4+ | primitive | mets | 12 | 6 | 0.0009 | 0.0112 | 5.405 | NA | -13.11, 70.68 | 31 | NA | 0.6820 | 0.9999 |
| Fig. S1 | CD8+ | primitive | mets | 12 | 6 | 0.0043 | 0.0344 | -0.250 | NA | -72.30, 37.45 | 36 | NA | >0.9999 | 0.9999 |
| Fig. S1 | CD163+ | primitive | mets | 12 | 6 | 0.1243 | 0.4232 | NA | 97.21 ± 171.7 | -300.4, 494.8 | NA | 0.5663 (7.805) | 0.5871 | 0.9999 |
| Fig. S1 | CD68+ | primitive | mets | 12 | 6 | <0.0001 | 0.5241 | 205.9 | NA | -55.56, 508.7 | 19 | NA | 0.1246 | 0.9999 |
| Fig. S1 | CTLA4+ | primitive | mets | 12 | 6 | 0.0026 | 0.2327 | -0.210 | NA | -1.980, 1.710 | 35 | NA | 0.9461 | 0.9999 |
| Fig. S1 | CD68-/  PD-L1+ | primitive | mets | 12 | 6 | <0.0001 | 0.1010 | 0.029 | NA | -1.160, 0.330 |  | NA | 0.5051 | 0.9999 |

All immunological markers evaluated in the study are reported irrespective of statistical significance. Markers included in the figures are identified by the corresponding figure, whereas markers not graphically represented are indicated as NDF (not displayed in figures). The distribution of continuous variables was assessed separately for Group 1 (Gr. 1) and Group 2 (Gr. 2) using the Shapiro–Wilk normality test, and the corresponding p-values are reported. For variables compatible with a normal distribution, comparisons were performed using the Welch’s unpaired t-test, and between-group differences were quantified as differences between means (mean difference). For non-normally distributed variables, comparisons were performed using the Mann–Whitney U test, and between-group differences were quantified using the Hodges–Lehmann estimator. All estimates represent Group 2 minus Group 1. For Mann–Whitney U comparisons, the 95% confidence interval (CI) refers to the Hodges–Lehmann estimate; for Welch’s t-test comparisons, it refers to the difference between means. Welch’s t statistics [Welch’s t(df)] are reported with corresponding degrees of freedom (df) in parentheses. Raw two-sided p-values were adjusted using the Benjamini–Hochberg procedure within each biologically defined family of comparisons to control the false discovery rate (FDR) at 5%. Statistical significance after correction was assessed using an FDR-adjusted q-value < 0.05. NA, not applicable.

**Supplementary Table 4.** CIBERSORTx relative scores of the immune populations detected in our cohort (*n*=12).

| **ID** | **B cells naive** | **B cells memory** | **Plasma cells** | **T cells CD8** | **T cells CD4 naive** | **T cells CD4 memory resting** | **T cells CD4 memory activated** | **T cells follicular helper** | **T cells regulatory (Tregs)** | **T cells gamma delta** | **NK cells resting** | **NK cells activated** | **Monocytes** | **Macrophages M0** | **Macrophages M1** | **Macrophages M2** | **Dendritic cells resting** | **Dendritic cells activated** | **Mast cells resting** | **Mast cells activated** | **Eosinophils** | **Neutrophils** |
| --- | --- | --- | --- | --- | --- | --- | --- | --- | --- | --- | --- | --- | --- | --- | --- | --- | --- | --- | --- | --- | --- | --- |
| **GIST_560** | 0.142 | 0.000 | 0.010 | 0.075 | 0.000 | 0.771 | 0.021 | 0.000 | 0.000 | 0.000 | 0.109 | 0.078 | 0.164 | 0.000 | 0.160 | 0.409 | 0.013 | 0.062 | 0.076 | 0.000 | 0.128 | 0.003 |
| **GIST_572** | 0.143 | 0.000 | 0.012 | 0.045 | 0.000 | 0.787 | 0.005 | 0.000 | 0.000 | 0.000 | 0.094 | 0.025 | 0.117 | 0.000 | 0.037 | 0.449 | 0.012 | 0.034 | 0.059 | 0.000 | 0.043 | 0.006 |
| **GIST_543** | 0.136 | 0.008 | 0.000 | 0.000 | 0.045 | 0.314 | 0.100 | 0.000 | 0.000 | 0.030 | 0.037 | 0.029 | 0.079 | 0.000 | 0.045 | 0.317 | 0.016 | 0.010 | 0.066 | 0.000 | 0.099 | 0.020 |
| **GIST_565** | 0.144 | 0.000 | 0.036 | 0.015 | 0.000 | 0.518 | 0.031 | 0.000 | 0.000 | 0.012 | 0.025 | 0.000 | 0.135 | 0.000 | 0.033 | 0.477 | 0.014 | 0.011 | 0.064 | 0.000 | 0.074 | 0.018 |
| **GIST_566** | 0.098 | 0.000 | 0.008 | 0.109 | 0.000 | 0.726 | 0.066 | 0.010 | 0.000 | 0.000 | 0.079 | 0.064 | 0.073 | 0.000 | 0.107 | 0.260 | 0.018 | 0.000 | 0.113 | 0.000 | 0.006 | 0.006 |
| **GIST_568** | 0.120 | 0.000 | 0.013 | 0.056 | 0.000 | 0.545 | 0.128 | 0.022 | 0.066 | 0.000 | 0.135 | 0.012 | 0.107 | 0.000 | 0.059 | 0.530 | 0.063 | 0.017 | 0.132 | 0.000 | 0.035 | 0.000 |
| **GIST_571** | 0.077 | 0.018 | 0.016 | 0.019 | 0.000 | 0.395 | 0.000 | 0.027 | 0.031 | 0.024 | 0.043 | 0.093 | 0.112 | 0.000 | 0.080 | 0.291 | 0.016 | 0.013 | 0.102 | 0.000 | 0.017 | 0.006 |
| **GIST_573** | 0.083 | 0.075 | 0.002 | 0.012 | 0.000 | 0.493 | 0.124 | 0.000 | 0.000 | 0.085 | 0.000 | 0.080 | 0.043 | 0.035 | 0.094 | 0.270 | 0.012 | 0.006 | 0.133 | 0.000 | 0.091 | 0.000 |
| **GIST_574** | 0.175 | 0.000 | 0.004 | 0.134 | 0.000 | 1.144 | 0.018 | 0.000 | 0.000 | 0.070 | 0.000 | 0.043 | 0.021 | 0.000 | 0.102 | 0.427 | 0.028 | 0.008 | 0.032 | 0.000 | 0.015 | 0.005 |
| **GIST_575** | 0.104 | 0.000 | 0.003 | 0.020 | 0.000 | 0.512 | 0.000 | 0.048 | 0.033 | 0.000 | 0.084 | 0.022 | 0.104 | 0.017 | 0.051 | 0.230 | 0.022 | 0.027 | 0.118 | 0.000 | 0.039 | 0.000 |
| **GIST_557** | 0.155 | 0.321 | 0.109 | 0.035 | 0.000 | 0.414 | 0.085 | 0.043 | 0.043 | 0.000 | 0.031 | 0.059 | 0.606 | 0.000 | 0.081 | 0.791 | 0.010 | 0.007 | 0.052 | 0.000 | 0.022 | 0.000 |
| **GIST_563** | 0.406 | 0.000 | 0.157 | 0.239 | 0.000 | 0.910 | 0.068 | 0.000 | 0.077 | 0.000 | 0.000 | 0.224 | 0.520 | 0.029 | 0.072 | 0.558 | 0.006 | 0.005 | 0.132 | 0.000 | 0.033 | 0.000 |
